# Cancer Metastasis Prediction and Genomic Biomarker Identification through Machine Learning and eXplainable Artificial Intelligence in Breast Cancer Research

**DOI:** 10.3390/diagnostics13213314

**Published:** 2023-10-26

**Authors:** Burak Yagin, Fatma Hilal Yagin, Cemil Colak, Feyza Inceoglu, Seifedine Kadry, Jungeun Kim

**Affiliations:** 1 Department of Biostatistics and Medical Informatics, Faculty of Medicine, Inonu University, Malatya 44280, Turkey; burak.yagin@inonu.edu.tr (B.Y.); cemil.colak@inonu.edu.tr (C.C.); 2 Department of Biostatistics, Faculty of Medicine, Malatya Turgut Ozal University, Malatya 44090, Turkey; feyza.inceoglu@ozal.edu.tr; 3 Department of applied Data science, Noroff University College, 4612 Kristiansand, Norway; seifedine.kadry@noroff.no; 4 Artificial Intelligence Research Center (AIRC), Ajman University, Ajman 346, United Arab Emirates; 5 Department of Electrical and Computer Engineering, Lebanese American University, Byblos 36, Lebanon; 6 Department of Software, Kongju National University, Cheonan 31080, Republic of Korea

**Keywords:** breast cancer metastasis, machine learning algorithms, genomic biomarkers, eXplainable artificial intelligence, SHAP

## Abstract

Aim: Method: This research presents a model combining machine learning (ML) techniques and eXplainable artificial intelligence (XAI) to predict breast cancer (BC) metastasis and reveal important genomic biomarkers in metastasis patients. Method: A total of 98 primary BC samples was analyzed, comprising 34 samples from patients who developed distant metastases within a 5-year follow-up period and 44 samples from patients who remained disease-free for at least 5 years after diagnosis. Genomic data were then subjected to biostatistical analysis, followed by the application of the elastic net feature selection method. This technique identified a restricted number of genomic biomarkers associated with BC metastasis. A light gradient boosting machine (LightGBM), categorical boosting (CatBoost), Extreme Gradient Boosting (XGBoost), Gradient Boosting Trees (GBT), and Ada boosting (AdaBoost) algorithms were utilized for prediction. To assess the models’ predictive abilities, the accuracy, F1 score, precision, recall, area under the ROC curve (AUC), and Brier score were calculated as performance evaluation metrics. To promote interpretability and overcome the “black box” problem of ML models, a SHapley Additive exPlanations (SHAP) method was employed. Results: The LightGBM model outperformed other models, yielding remarkable accuracy of 96% and an AUC of 99.3%. In addition to biostatistical evaluation, in XAI-based SHAP results, increased expression levels of TSPYL5, ATP5E, CA9, NUP210, SLC37A1, ARIH1, PSMD7, UBQLN1, PRAME, and UBE2T (*p* ≤ 0.05) were found to be associated with an increased incidence of BC metastasis. Finally, decreased levels of expression of CACTIN, TGFB3, SCUBE2, ARL4D, OR1F1, ALDH4A1, PHF1, and CROCC (*p* ≤ 0.05) genes were also determined to increase the risk of metastasis in BC. Conclusion: The findings of this study may prevent disease progression and metastases and potentially improve clinical outcomes by recommending customized treatment approaches for BC patients.

## 1. Introduction

Breast cancer (BC) is one of the most prevalent and life-threatening malignancies that profoundly impact women on a global scale, contributing significantly to the burden of cancer-related morbidity and mortality [1]. Despite remarkable strides made in the realms of early detection and therapeutic interventions, BC continues to pose a formidable challenge to public health systems and healthcare providers worldwide [2].

Metastasis, defined as the dissemination of cancer cells from the primary tumor site to distant organs or tissues, represents a critical turning point in the disease’s progression. Metastatic BC, in particular, assumes a grim prominence due to its association with heightened levels of morbidity and mortality [3]. Hence, the development of precise predictive models and the identification of genomic biomarkers assume paramount importance, offering potential solutions to the pressing need for early detection and effective management of this life-threatening condition [4]. BC prognosis may be generally worse when metastases are present with respect to clinical evaluation. Additionally, the survival of BC patients relies on many predictors, including the stage/grade of the BC [5]. 

Genomic biomarkers are pivotal in predicting and detecting BC metastasis, offering insights into the molecular complexities behind it. They facilitate early detection and assess the tumor’s metastatic potential. Identifying specific metastasis-associated genes helps identify high-risk patients for personalized treatment. Furthermore, these biomarkers unveil the underlying mechanisms of metastasis, paving the way for targeted therapies aimed at preventing its progression. This wealth of information not only enhances patient care but also fuels ongoing research into more effective interventions, ultimately improving outcomes for those affected by BC metastasis [6,7,8].

In recent years, machine learning (ML) and artificial intelligence (AI) approaches have revolutionized the field of medical research by offering new ways to understand complex diseases [9]. ML algorithms have demonstrated remarkable potential in the analysis of large genomic datasets, thus facilitating the discovery of predictive biomarkers and the development of innovative prognostic models [10]. Applications of ML in the field of radiomics have also begun to attract ever more attention recently. Clinical quality management systems have been improved with models to be developed beyond diagnosis and treatment. Increasing data suggest that ML and radiomics can be used to improve tumor characterization, such as some tumor molecular features, association with tumor spread, and prognosis [11].

Furthermore, the integration of eXplainable AI (XAI) techniques in the field of medical research serves to elucidate the decision-making processes inherent in ML algorithms. This increased transparency not only increases the interpretability of these algorithms, but also strengthens their overall reliability, especially in the complex environment of medical applications. XAI has the potential to advance a more comprehensive understanding of BC metastasis and the identification of genomic biomarkers, thereby opening new avenues for transformative advances in BC research and patient care. Additionally, XAI helps with drug discovery in BC, personalizing early treatment plans [12,13]. XAI provides transparency in decision-making, making the rationale behind diagnosis and treatment recommendations clearer to patients and clinicians [14,15]. 

However, more evidence is needed regarding the poor performance of predictive models in BC metastasis, and the difficulties in understanding complex model predictions. This study used an innovative methodology that combines the power of ML and XAI to obtain highly accurate predictions of BC metastasis. Moreover, this approach revealed important genomic biomarkers that are intricately linked to the progression of metastasis. By leveraging the computational power of ML and the interpretability of XAI, our aim is not only to increase the accuracy of metastasis prediction but also to decipher potential genomic signatures in prognosis and treatment strategies for BC patients. This multidisciplinary approach represents an important step towards improving BC patient care.

## 2. Materials and Methods

### 2.1. Data Source and Selection Criteria

The gene expression data for this study were sourced from the National Center for Biotechnology Information’s Gene Expression Omnibus (NCBI GEO) database [16]. A total of 98 primary BC samples was strategically selected based on specific clinical criteria (all patients included in this study were categorized as ”sporadic”, were lymph node-negative, and were under the age of 55 at the time of diagnosis). Of these, 34 samples were from patients who developed distant metastases within a 5-year follow-up period, while 44 were from patients who remained disease-free for at least 5 years post-diagnosis. The Inonu University Health Sciences Non-Interventional Clinical Research Ethics Committee approved this study (approval number: 2023/5043).

### 2.2. Sample Preparation and RNA Isolation

For each selected patient, 5 mg of total RNA was meticulously isolated from instant-frozen tumor tissue samples. This RNA served as the foundation for synthesizing complementary RNA (cRNA), which was subsequently used for microarray analysis. A reference cRNA pool was generated by amalgamating equal quantities of cRNA from each sporadic carcinoma sample, serving as a standard baseline for subsequent analyses. 

### 2.3. Microarray Hybridization and Data Normalization

Two separate hybridizations were conducted for each tumor sample using a state-of-the-art fluorescent dye inversion technique. These hybridizations were performed on microarrays containing approximately 25,000 human genes, synthesized through advanced inkjet technology. Following the hybridization process, the fluorescent intensities of the scanned microarray images were quantitatively measured. These raw intensity values were then subjected to a rigorous normalization and correction process to derive the relative transcript abundance of each gene, expressed as an intensity ratio in comparison to the reference cRNA pool.

### 2.4. Biostatistical Data Analysis

The conformity of the variables to the normal distribution was examined by visual (histogram and probability graphs) and analytical (Shapiro–Wilk test) methods. Because the data did not show a normal distribution, the genomic data were summarized using the interquartile range (IQR) together with the median, and the Mann–Whitney U test was used for comparisons between the two groups. A Spearman rank correlation graph was drawn to examine the relationships between genomic biomarkers. A *p*-value of ≤0.05 was considered statistically significant in all results. Statistical analyses were performed using a SPSS 28.0 (IBM Corp., Armonk, NY, USA) package program.

### 2.5. ML and XAI Approach

#### 2.5.1. Data Preprocessing

In this study, robust standardization was first applied to the data to convert the inputs to comparable scales and purify the effects of outliers on the modelling [17]. The elastic net feature selection method was applied to the data to reduce the size of the genomic data and identify a small number of biomarkers. The elastic net is an orchestration method used in ML and statistical modeling to handle multicollinearity and feature selection tasks. The elastic net is a valuable method for datasets with a large number of related features that can handle high-dimensional datasets. The method balances sparsity and model interpretability by combining L1 and L2 regularization, performing feature selection and coefficient narrowing [18,19].

#### 2.5.2. ML Algorithms Used for BC Metastasis Prediction

Light Gradient Boosting Machine (LightGBM): A gradient boost-based algorithm, LightGBM is a fast and efficient algorithm for ML tasks. Thus, it is often used for ML tasks on large-scale datasets. This algorithm uses a “Gradient-based One-Side Sampling” (GOSS), which reduces memory usage and considerably speeds up model training. It also natively supports categorical features and implements histogram-based splitting for faster computation [20,21].

Categorical Boosting (CatBoost): CatBoost, a gradient boost-based algorithm, is fitted with built-in processing of categorical features, eliminating the need for manual coding. CatBoost uses a sequential boosting method that considers the order of categorical variables during the boosting process. In addition, it uses symmetric decision trees to improve generalization performance and includes various techniques to optimize sequencing in the learning process [22,23].

Extreme Gradient Boosting (XGBoost): XGBoost is one of the most widely used gradient boosting algorithms, providing high performance and flexibility. This algorithm supports various loss functions and customization options. XGBoost uses a more regular model formalization compared to the traditional gradient boosting method and includes techniques such as weighted quantitative plotting for approximate tree learning [24,25].

Gradient Boosting Trees (GBT): GBT is an algorithm that uses decision trees as core learners and expresses the general concept of gradient reinforcement. GBTs work iteratively to build a collection of weak decision trees, and each subsequent tree is trained to correct the errors of previous trees. GBTs can use different loss functions, but the most widely used method relies on squared error loss for regression tasks [26,27].

Ada Boosting (AdaBoost): AdaBoost is an ensemble learning method that combines multiple weak classifiers to create a strong classifier. It highlights difficult samples by adjusting the weights of misclassified samples at each iteration. Misclassified samples are given higher weights, allowing greater focus on these samples in subsequent iterations. AdaBoost can work with any classification algorithm as its base estimator, and decision logs (single-level decision trees) are generally preferred. It adjusts the weights of the misclassified samples to improve the model’s performance and makes the weak classifiers a strong ensemble [28,29].

#### 2.5.3. Performance Evaluation Metrics

The performance of the ML models was evaluated by calculating the accuracy, F1 score, precision, recall, area under the ROC curve (AUC), and Brier score, and the results were compared [30,31]. Below is a brief description of each metric:

Accuracy: Accuracy measures the overall accuracy of the model’s predictions and is the most fundamental metric in classification tasks. It can be calculated by dividing the correctly predicted samples by the total number of samples in the data set.

F1 score: The F1 score is a harmonic mean of precision and recall. It provides a single measurement that balances both precision and recall. The F1 score is often quite important when the dataset is unbalanced.

Precision: Precision measures the proportion of positive cases correctly predicted out of all positively predicted cases. It is calculated as the ratio of true positives to the sum of true positives and false positives.

Recall: Recall, also known as the sensitivity or true positive rate, measures the proportion of correctly predicted positive samples out of all true positive samples. It is calculated as the ratio of true positives to the sum of true positives and false negatives.

AUC: AUC measures the model’s ability to distinguish between positive and negative classes. A higher AUC value indicates better model performance.

Brier score: The Brier score is a convenient scoring rule used to evaluate the accuracy of probabilistic predictions. It measures the mean squared difference between predicted probabilities and actual results. Lower Brier scores indicate better calibration and accuracy of the model.

#### 2.5.4. XAI Approach and Feature Importance

ML models are often called “black boxes” because it can be challenging to understand why an algorithm produces accurate predictions for a given cohort of patients. This study used the model with optimal performance for the final estimation, and the SHAP method was applied to explain the model’s decisions. The relevance of features in the final predictive model was prioritized to find important metastasis biomarkers in the patient group.

##### SHapley Additive exPlanations (SHAP)

SHAP is a unified framework for interpreting ML models. Compared to other XAI methods, SHAP can provide both local and global interpretability simultaneously and is based on a solid theoretical foundation. It provides a game-theoretic approach to attributing the contribution of each feature to the prediction made by a model. The Shapley value measures the average marginal contribution of each trait across all possible coalitions. SHAP is a method that also provides a way to evaluate the importance of features by measuring their contribution to the prediction. The method allows us to interpret the contribution of individual characteristics for a given prediction. By attributing each feature, it provides the difference between the model’s estimate and the baseline estimate in local annotations. SHAP can also provide an overview of feature importance across the entire dataset. It quantifies the overall effect of each feature on the model’s predictions by aggregating the Shapley values of each feature across multiple samples. The SHAP method provides a comprehensive understanding of feature significance and individual feature contributions, assisting model debugging, validation, and decision-making. In addition, SHAP differs from other ML models’ ability to evaluate whether each input feature positively or negatively impacts the prediction [32,33,34].

## 3. Results

The initial dataset contained expression levels for a substantial number of 24,481 genes, presenting a challenge of high dimensionality that is often encountered in genomics research. To address this issue, we employed the elastic net feature selection algorithm, a method known for its capability to manage multicollinearity while simultaneously performing variable selection. Subsequent to the application of the elastic net algorithm, we were able to identify a refined set of 18 genes that emerged as strong biomarker candidates for BC metastasis. The elastic net model reduced the size of the data set by 99.93%. These genes were selected based on their statistical significance and potential biological relevance to metastatic progression.

Descriptive statistics and effect size estimates for these 18 biomarker-candidate genes are comprehensively reported in Table 1. Notably, the *p*-values for all selected genes were found to be statistically significant with *p* ≤ 0.05, reinforcing the robustness of our feature selection process and the putative relevance of these genes in the context of BC metastasis. Our analysis further revealed that among the 18 selected genes, TSPYL5 exhibited the most substantial effect size (ES: 0.306). This suggests that TSPYL5 serves as a potent discriminator between the metastasis-positive and metastasis-negative groups, thereby warranting further investigation as a potential therapeutic target or diagnostic biomarker (Table 1).

**Figure 1:**
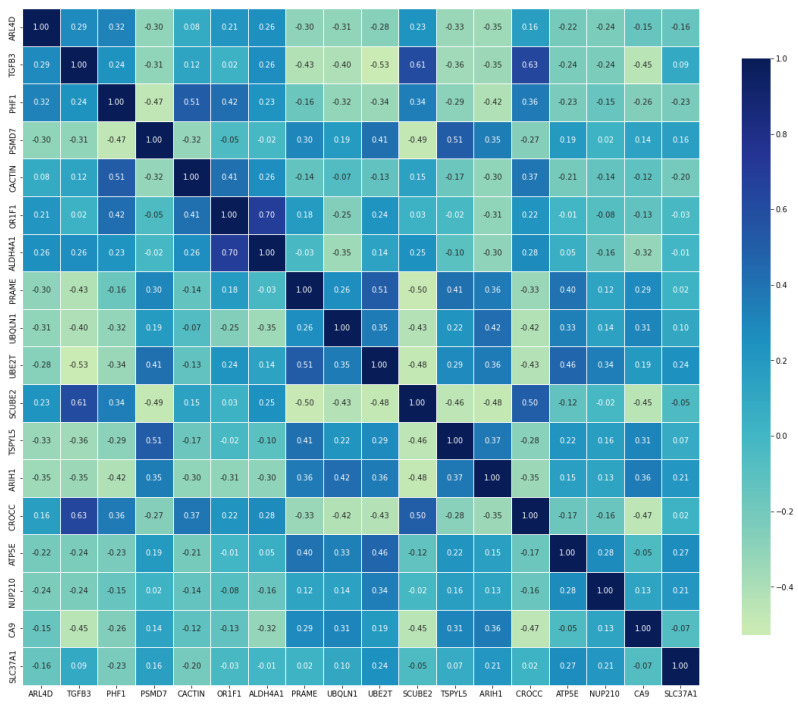
Correlation heatmap representation.

In this investigation, Spearman correlation analysis was applied to explore the interplay among 18 genes identified as potential BC metastasis biomarkers, visualized through a heatmap for clarity (Figure 1).

Table 2 comprehensively delineates the performance metrics—namely, accuracy, F1 score, precision, recall, AUC, and Brier score—for five ML algorithms: LightGBM, CatBoost, XGBoost, GBT, and AdaBoost. These metrics were meticulously evaluated to provide a holistic understanding of each model’s predictive capabilities for BC metastasis. Following scrutiny of the performance metrics, LightGBM emerged as the most efficacious algorithm, significantly outperforming the other four models. It achieved an impressive accuracy of 96% and an AUC value of 99.3%, metrics that are generally considered gold standards in classification tasks. Equally noteworthy is the Brier score, a lesser-known but highly informative metric for evaluating the predictive reliability of probabilistic models. The Brier score for the LightGBM model was 0.024, falling well below the generally accepted threshold of 0.25. This suggests that not only is the model highly accurate, but it is also well calibrated, providing a high level of confidence in its predictive probabilities. The superior performance of LightGBM can be attributed to its robust handling of high-dimensional, imbalanced datasets, a common challenge in medical research. Additionally, the AUC value of 99.3% indicates an almost perfect ability of the model to discriminate between metastasis-positive and metastasis-negative cases, further solidifying its utility in clinical settings (Table 2).

**Figure 2:**
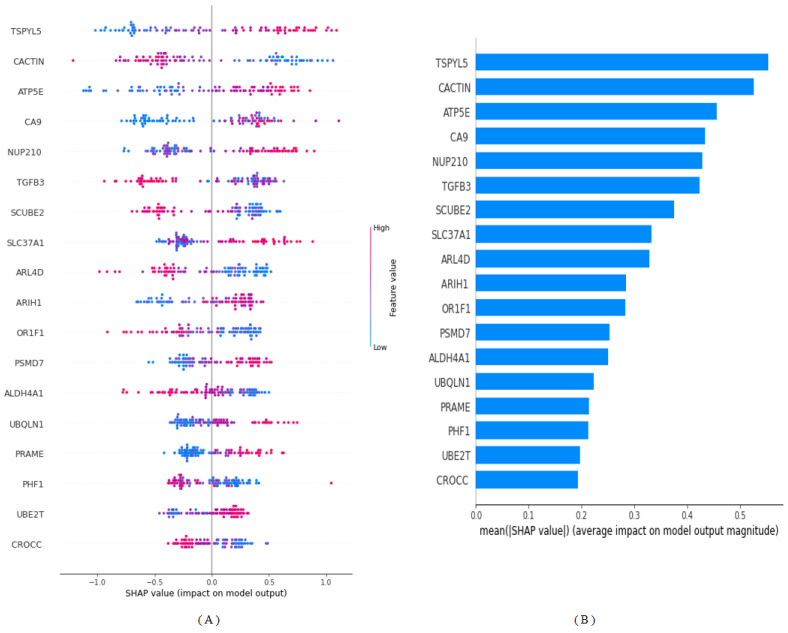
LightGBM model interpretation. (**A**): Using the final model, we rank the relevance of the top 18 biomarker genes regarding stability and interpretation. (**B**): The mean order of importance of the first 18 biomarker genes (|SHAP value|); the higher the SHAP value of a feature is provided, the more likely it is the patient will be BC metastasis-positive.

Figure 2 offers a detailed visual representation of SHAP annotations, elucidating the positive or negative contributions of candidate biomarker genes in the context of our optimal ML model—LightGBM. SHAP values serve as an indispensable tool in the interpretability of complex ML models, especially in critical domains such as healthcare. In this analysis, a positive SHAP value signifies a positive contribution toward the target variable (metastasis risk), while a negative SHAP value indicates an inverse relationship. These values are arranged in descending order to prioritize their influence on the model’s output. According to the SHAP analysis, the genes TSPYL5, CACTIN, and ATP5E emerge as the top contributors to BC metastasis prediction. Their prominent roles in the model underscore their potential importance as therapeutic targets or early-warning biomarkers. The graphical representation employs a color-coded scheme based on normalized gene expression levels. A shift toward blue represents a decrease in gene expression levels, while a shift toward pink indicates an increase. The analysis reveals that higher expression levels of TSPYL5, ATP5E, CA9, NUP210, SLC37A1, ARIH1, PSMD7, UBQLN1, PRAME, and UBE2T are associated with an increased risk of BC metastasis. Furthermore, lower levels of expression of CACTIN, TGFB3, SCUBE2, ARL4D, OR1F1, ALDH4A1, PHF1, and CROCC (*p* ≤ 0.05) genes were also determined to increase the risk of metastasis in BC (Figure 2).

Upon scrutinizing the normalized SHAP values presented in Table 3, we identify a set of five genes—TSPYL5, CACTIN, ATP5E, CA9, and NUP210—as the most salient risk factors contributing to the likelihood of BC metastasis. These genes contribute distinct percentages to the model’s prediction of metastasis risk. Specifically, TSPYL5 contributes 9.2%, CACTIN accounts for 8.8%, ATP5E for 7.6%, CA9 for 7.2%, and NUP210 for 7.1%. The substantial contributions of these genes point to their potential as critical biomarkers for early detection or as novel targets for therapeutic interventions. Their varying but significant percentages indicate a complex interplay of genetic factors that need to be considered in the development of more personalized and effective treatment regimens for BC patients.

## 4. Discussion

In this study, the 18 most important genes (TSPYL5, CACTIN, ATP5E, CA9, NUP210, TGFB3, SCUBE2, SLC37A1, ARL4D, ARIH1, OR1F1, PSMD7, ALDH4A1, UBQLN1, PRAME, PHF1, UBE2T, and CROCC) associated with BC metastasis were identified after elastic net feature selection due to its ability to manage both multicollinearity and feature selection efficiently [35]. Among the algorithms evaluated in the study, which applied a versatile ML approach to predicting BC metastasis, LightGBM achieved an impressive accuracy rate of 96%. This superior performance highlights the potential and robustness of advanced ML algorithms for complex and high-dimensional datasets commonly encountered in medical research, especially genomics. Identification of important predictive genes (TSPYL5, CACTIN, ATP5E, CA9, and NUP210) opens up new avenues for early diagnosis and targeted therapies. The potential to tailor treatment regimens to an individual’s unique genetic profile holds promise for significantly improving clinical outcomes. It shifts the paradigm from a ”one size fits all” approach to a more personalized healthcare model. Furthermore, SHAP results reveal that higher expression levels of TSPYL5, ATP5E, CA9, NUP210, SLC37A1, ARIH1, PSMD7, UBQLN1, PRAME, and UBE2T are associated with an increased risk of BC metastasis. Conversely, lower expression levels of CACTIN, TGFB3, SCUBE2, ARL4D, OR1F1, ALDH4A1, PHF1, and CROCC appear to mitigate this risk. In addition to accuracy and interpretability, calibration of predictive models is also crucial to their clinical applicability. The Brier score, an often overlooked metric, was calculated to evaluate the model’s predictive performance. A score of 0.024, well below the generally accepted threshold of 0.25, indicates that the model is well calibrated [36]. This adds another layer of confidence in the usefulness of the model in the clinical setting. Our work addresses an important gap in the existing literature by harmoniously integrating advanced prediction algorithms with explainability elements. While previous studies often prioritize either prediction accuracy or model interpretability, our approach manages to strike a balance between the two [37,38]. This harmonization is vital to the practical application of the model, as clinicians need models that are both accurate and interpretable for effective decision-making.

The current study encompassed the most important genes associated with BC metastasis. As reported in this article, the TSPYL5 gene, situated at the 8q22.1 locus, encodes the testis-specific Y-encoded-like protein 5, as highlighted in previous research [39]. Notably, heightened expression of this gene has emerged as a significant factor in breast oncogenesis and has been linked to an unfavorable prognosis. This effect is attributed to its capacity to suppress the function of the tumor suppressor protein P53, a pivotal guardian of genomic integrity and cell cycle regulation. While our understanding of the precise role of TSPYL5 in the context of cancer remains somewhat limited, earlier studies have posited that it may operate as a transcription factor for a spectrum of genes associated with ER-positive BC [40]. This implies that TSPYL5 may exert regulatory influence over genes that are crucial in the context of estrogen receptor-positive BC, a subtype that constitutes a substantial proportion of BC cases. Consequently, further exploration of the molecular mechanisms underlying TSPYL5’s involvement in BC is warranted, as it may yield critical insights into the development and progression of this disease, potentially paving the way for targeted therapeutic strategies for ER-positive BC patients [41]. According to the SHAP results, TSPYL5 was determined as the most important gene for BC metastasis prediction in our study.

One of the most well-known genes linked to hypoxia in tumor cells is CA9, which is rapidly and significantly increased in hypoxic environments. A family of zinc metalloenzymes is known as CAs. In another study in the literature, the authors reported that the CA9 gene was detectable in breast tumors and was associated with resistance to both adjuvant chemotherapy and endocrine therapy [42]. In the current study, we found that the CA9 gene is important in BC metastasis prediction.

The role of ALDH1A1 in the retinoic acid signaling pathway is crucial, as it governs the self-renewal and differentiation processes of normal stem cells and also holds significant implications in cancer progression. Liu et al. [43] have underscored the importance of ALDH1A1 mRNA expression levels within tumor tissues, suggesting that it could serve as an independent predictor for a favorable outcome in triple-negative BC. This finding implies that monitoring ALDH1A1 expression may offer valuable insights into the prognosis of triple-negative BC cases.

NPC proteins have lately been linked to a number of malignancies and developmental abnormalities [44,45]. The NPC protein gene Nup210, which affects the mechanical response, focal adhesion, and cell migration without affecting nucleocytoplasmic transport, has been found to be responsive to mechanical signals in the extracellular microenvironment and to promote lung metastasis in mouse models of breast cancer [46]. In another study, the authors identified the NUP210 gene as a potential metastasis susceptibility gene for human ER+ BC patients [46]. NUP210 was among the five most important genes in the metastasis prediction model in the current study.

Epping et al. [47] have underscored the significance of PRAME expression as a prognostic marker in BC patients. Their study results indicated that PRAME serves as an independent predictor of a shortened metastasis-free interval, particularly in patients not receiving adjuvant chemotherapy. Furthermore, PRAME expression has been associated with tumor grade and negative estrogen receptor status, signifying its potential utility in delineating high-risk BC cases.

## 5. Limitations

One of the limitations of the current study is that the prediction of BC metastasis requires further validation. Larger and more diverse datasets are required to confirm the generalizability of findings. The exploration of other ML techniques, such as neural networks, could yield even more nuanced insights into BC metastasis. Furthermore, the incorporation of additional clinical variables could enrich the model, enhancing its predictive power and clinical relevance.

## 6. Conclusions

In conclusion, the study makes a seminal contribution to BC research by achieving a symbiotic balance between predictive accuracy and model interpretability. It sets a new benchmark for future research in this critical healthcare domain by offering not only a robust predictive model but also unprecedented insights into the genomic variables influencing BC metastasis. By bridging the gap between high predictive accuracy and model interpretability, our study paves the way for more effective and personalized healthcare solutions in the fight against BC.

## Figures and Tables

**Table 1:** Descriptive statistics with respect to the study groups.

Gene *	BC Metastasis Status		U-Value	*p*-Value **	ES
	Non-Metastasis	Metastasis			
ARL4D	0.401 (1.028)	−0.198 (0.477)	1093	<0.001	0.143 (medium)
TGFB3	0.38 (0.994)	−0.248 (0.754)	1093	<0.001	0.164 (medium)
PHF1	0.409 (0.916)	−0.221 (0.505)	788	<0.001	0.173 (medium)
PSMD7	−0.228 (0.67)	0.458 (0.808)	887	<0.001	0.188 (medium)
CACTIN	0.248 (0.74)	−0.36 (0.748)	865	<0.001	0.213 (medium)
OR1F1	0.404 (1.237)	−0.128 (0.471)	932.5	<0.001	0.143 (medium)
ALDH4A1	0.312 (0.713)	−0.412 (0.962)	982.5	<0.001	0.194 (medium)
PRAME	−0.072 (0.226)	0.744 (1.158)	1086.5	<0.001	0.135 (medium)
UBQLN1	−0.381 (0.755)	0.262 (1.06)	1042	<0.001	0.17 (medium)
UBE2T	−0.329 (0.923)	0.327 (0.635)	986	<0.001	0.176 (medium)
SCUBE2	0.245 (0.484)	−0.576 (0.735)	915	<0.001	0.212 (medium)
TSPYL5	−0.305 (0.599)	0.51 (0.759)	961.5	<0.001	0.306 (large)
ARIH1	−0.396 (0.935)	0.331 (0.792)	844	<0.001	0.197 (medium)
CROCC	0.455 (0.77)	−0.291 (0.706)	1022	<0.001	0.191 (medium)
ATP5E	−0.401 (0.818)	0.283 (1.057)	1013	<0.001	0.196 (medium)
NUP210	−0.094 (0.532)	0.468 (0.958)	1075	0.001	0.117 (medium)
CA9	−0.177 (0.617)	0.379 (1.171)	852	<0.001	0.176 (medium)
SLC37A1	−0.121 (0.872)	0.338 (1.085)	1051	0.002	0.0977 (medium)

*: Gene expression levels are summarized as ‘Median (IQR)’; **: Mann–Whitney U test; ES: effect size; BC: Breast cancer.

**Table 2:** Results of ML models performance metrics for BC metastasis prediction.

Model	Accuracy	F1 Score	Precision	Recall	AUC	Brier Score
LightGBM	96	96.8	100	93.8	99.3	0.024
CatBoost	84	86.7	92.9	81.2	85.1	0.057
XGBoost	92	93.8	93.8	93.8	97.9	0.026
GBT	80	82.8	92.3	75	94.4	0.081
AdaBoost	88	90.9	88.2	93.8	93.1	0.077

LightGBM: light gradient boosting machine; CatBoost: categorical boosting; XGBoost: extreme gradient boosting; GBT: gradient boosting tree; AdaBoost: Ada boosting; AUC: area under the curve.

**Table 3:** Contribution of the genes to BC metastasis prediction.

Gene Name	Importance Score
TSPYL5	0.092
CACTIN	0.088
ATP5E	0.076
CA9	0.072
NUP210	0.071
TGFB3	0.070
SCUBE2	0.062
SLC37A1	0.055
ARL4D	0.055
ARIH1	0.047
OR1F1	0.047
PSMD7	0.042
ALDH4A1	0.042
UBQLN1	0.037
PRAME	0.035
PHF1	0.035
UBE2T	0.032
CROCC	0.032

## Data Availability

Data are available for research purposes upon reasonable request to the corresponding author.
